# Relationships of Bone Mineral Variables with Body Composition, Blood Hormones and Training Volume in Adolescent Female Athletes with Different Loading Patterns

**DOI:** 10.3390/ijerph18126571

**Published:** 2021-06-18

**Authors:** Vita Tamolienė, Liina Remmel, Rita Gruodyte-Raciene, Jaak Jürimäe

**Affiliations:** 1 Institute of Sports Science and Innovations, Lithuanian Sports University, LT-44221 Kaunas, Lithuania; Vita.Karvelyte@lsu.lt; 2 Institute of Sport Sciences and Physiotherapy, Faculty of Medicine, University of Tartu, 50090 Tartu, Estonia; liina.remmel@ut.ee; 3 Department of Physical and Social Education, Lithuanian Sports University, LT-44221 Kaunas, Lithuania; Rita.Gruodyte@lsu.lt

**Keywords:** adolescent athletes, bone mineral accrual, training volume, hormones, appendicular muscle mass

## Abstract

The aim of this investigation was to determine the relationships of areal bone mineral density (aBMD) and content (BMC) with body composition, blood hormone and training load variables in adolescent female athletes with different loading patterns. The participants were 73 healthy adolescent females (14–18 years), who were divided into three groups: rhythmic gymnasts (RG; *n* = 33), swimmers (SW; *n* = 20) and untrained controls (UC; *n* = 20). Bone mineral and body compositional variables were measured by dual-energy X-ray absorptiometry, and insulin-like growth factor-1 (IGF-1), estradiol and leptin were analyzed from blood samples. In addition, aerobic performance was assessed by a peak oxygen consumption test. No differences (*p* > 0.05) in weekly training volume were observed between rhythmic gymnasts (17.6 ± 5.3 h/week) and swimmers (16.1 ± 6.9 h/week). Measured areal bone mineral density and bone mineral content values were higher in rhythmic gymnasts compared with other groups (*p* < 0.05), while no differences (*p* > 0.05) in measured bone mineral values were seen between swimmers and untrained control groups. Multiple regression models indicated that IGF-1 alone explained 14% of the total variance (R^2^ × 100) in lumbar spine aBMD, while appendicular muscle mass and training volume together explained 37% of the total variance in femoral neck BMC in the rhythmic gymnast group only. In swimmers, age at menarche, estradiol and appendicular muscle mass together explained 68% of the total variance in lumbar spine BMC, while appendicular muscle mass was the only predictor and explained 19 to 53% of the total variance in measured bone mineral values in untrained controls. In conclusion, adolescent rhythmic gymnasts with specific weight-bearing athletic activity present higher areal bone mineral values in comparison with swimmers and untrained controls. Specific training volume together with appendicular muscle mass influenced cortical bone development at the femoral neck site of the skeleton in rhythmic gymnasts, while hormonal values influenced trabecular bone development at the lumbar spine site in both athletic groups with different loading patterns.

## 1. Introduction

Bone health has become a concern in modern society as osteoporosis is the most common metabolic skeletal disease in humans and is major public health concern. Adolescence is a period of rapid bone mineral accrual and is a crucial time to optimize peak bone mass for osteoporosis prevention later in life [1,2,3]. Among the variables capable of affecting bone health, hormonal [4,5] and mechanical [6,7] factors that include body composition [8,9] and physical activity [10,11] play an important role in bone mineral accrual during adolescence. Accordingly, physical exercise is highly beneficial to increase areal bone mineral density (aBMD) and bone mineral content (BMC) during adolescence [12]. These beneficial effects of physical exercise are primarily linked to the mechanisms of mechanical loading either from the impact with the ground, such as various jumps and sprints and/or from skeletal muscle contractions, such as during strength training, but the relative importance of these two sources has not yet been exactly determined [6,13]. The nature of the stimuli is affected by the type, intensity, frequency and duration of the physical exercise [11]. Therefore, the intensity of the stimulus seems to be more important than its length [14], which explains why not all types of physical exercise have the same beneficial effect on bone [10,14]. In addition to mechanical loading factors related to physical exercises, body fat mass (FM) and lean body mass (LBM) contribute to bone development by increasing compressive forces during skeletal loading [6]. There is substantial evidence to support the view that both FM and LBM are both positively related to specific aBMD and BMC values in female adolescents with different physical activity patterns [4,5]. The effects of exercise training on bone health in athletes vary, from high-impact weight-bearing activities such as gymnastics to non-weight-bearing activities such as swimming [7,10]. While the high-impact mechanical loading of gymnastics training is especially osteogenic and increases bone mineral accrual during adolescence [6], systematic swimming training has no effect on bone mineral accrual and is not considered as an osteogenic sport [13]. In addition, different sport training variables, such as previous length of practice [7,15] and weekly training volume [8,16] may also positively affect bone health during adolescence. In contrast, excessive exercise stress may have detrimental effects on bone health in adolescent female athletes, which is accompanied by decreases in hormonal [17] and body FM [6] values.

Athletic activity has a direct effect on bone mass accumulation via mechanical loading [7,10], while an indirect effect is generated via hormonal regulation [6,18]. Sport training also stimulates different body composition tissues that consequently increase mechanical loading of specific athletic activities [8,12] and produce specific muscle- and adipose-tissue derived cytokines [10,19] that influence bone mass accumulation in highly trained female adolescent athletes. Specifically, bone mineral accrual in girls may be especially promoted by the increase in estrogens together with insulin-like growth factor-1 (IGF-1) concentrations [4,20], which correspond to the timing of menarche and peak bone mass acquisition [21]. In contrast, heavy athletic activity in states of negative energy homeostasis may exert an inhibitory effect on sex hormones in adolescent female athletes [6], and hypoestrogenism negatively affects bone mineral accrual by increasing bone resorption and decreasing bone formation markers in amenorrheic adolescent athletes [17]. In addition, FM together with specific adipose tissue-derived adipokines, including leptin concentration, have been related to bone mineral accrual in adolescent female athletes [5,22]. However, chronic athletic activity may decrease FM together with circulating leptin levels [23], and the impact of lowered leptin on bone mineral accrual in the presence of elevated energy expenditure and reduced FM is not entirely clear in adolescent female athletes [22,24]. It appears that the effect of leptin on bone mineral accrual is likely multifactorial and may involve other hormones, such as estradiol and IGF-1, in addition to its direct actions on bone health in adolescent athletes [6,18]. Accordingly, the impact of specific hormones on bone mineral accrual in heavily exercising adolescent female athletes is not entirely clear and may depend on specific athletic activity.

The purpose of this investigation was to describe bone mineral characteristics in adolescent female athletes participating in two intense athletic activity patterns (rhythmic gymnastics as a high-impact weight-bearing sport and swimming as a non-weight-bearing sport) that generate different mechanical loading patterns on bone. Another aim was to determine the relationships of bone mineral values with body composition, blood hormone and training load variables in the studied groups.

## 2. Materials and Methods

### 2.1. Participants and Research Design

A total of 73 healthy adolescent females with ages ranging from 14 to 18 years participated in this study. Before entering the study, participants completed medical and training activity questionnaires. Information about age at menarche, changes in the menstrual cycle, past or present diseases and any kind of medication, vitamin or mineral supplement was collected [25]. None of the participants was receiving any medications or had a history of bone or renal diseases. No restrictions were placed on dietary intake, and participants consumed their everyday diet [5,26]. According to their training activity pattern, participants were divided into three groups: rhythmic gymnasts (RG; *n* = 33), swimmers (SW; *n* = 20) and untrained controls (UC; *n* = 20). All athletes were recruited from local training groups and were competing at the national or international level. RG and SW athletes had trained regularly for the last 10.3 ± 0.9 and 8.8 ± 1.4 years, with a mean weekly training volume of 17.6 ± 5.3 and 16.1 ± 6.9 h/week, respectively. Adolescents in the UC group took part only in compulsory physical education classes at school and did not attend any sport trainings after school. All SW and UC adolescent females were eumenorrheic, while 22 participants in the RG group were eumenorrheic and 11 had secondary amenorrhea. Menstruating adolescent females were examined during the follicular phase, where the blood sample was taken between days 7 and 11 from the onset of menstruation [25].

The study design, purpose and possible risks were explained to the participants and their parents, who gave their written informed consent before entering the study. The study protocol was approved by the Medical Ethics Committee of the University of Tartu, Estonia, and was conducted in accordance with the Declaration of Helsinki. Participants underwent an observational cross-sectional examination. Measurements of the current investigation included anthropometry, body composition, bone mineral, peak oxygen consumption and blood analyses.

### 2.2. Measurements

#### 2.2.1. Body Composition and Bone Mineral Parameters

Body height was measured to the nearest 0.1 cm using Martin’s metal anthropometer (GMP Anthropological Instruments, Zurich, Switzerland). Body mass was measured to the nearest 0.05 kg using medical scales (A&D Instruments Ltd., Abingdon, UK). Body mass index (BMI) was calculated as a ratio of body mass to the height squared (kg/m^2^). Body composition and bone mineral parameters were measured by dual-energy X-ray absorptiometry (DXA) using the DPX-IQ densitometer (Lunar Corporation, Madison, WI, USA) equipped with proprietary software, version 3.6. Participants were scanned in light clothing while lying flat on their backs with arms at their sides. Whole body FM (in % and kg) and LBM (in kg) were measured, and appendicular skeletal muscle mass as a surrogate marker of muscle mass was calculated as the sum of the lean soft tissue masses in the arms and legs in kg [27,28]. In addition, femoral neck (FN) aBMD (in g/cm^2^) and FN BMC (in g) were measured to describe cortical bone, and lumbar spine (LS; L2-L4) aBMD (in g/cm^2^) and LS BMC (in g) were measured to describe trabecular bone [4]. All DXA measurements and results were evaluated by the same examiner. The precision of measurement expressed as a coefficient of variation (CV) was less than 2% for all body composition and bone mineral measurements [27].

#### 2.2.2. Aerobic Performance

Maximal aerobic performance was determined by a stepwise incremental exercise test until volitional exhaustion using an electrically braked bicycle ergometer (Corival V3; Lode, the Netherlands) [25]. The initial work rate was 40 W, and the stage increment was 35 W after every 3 min until maximal voluntary exhaustion was reached. Pedaling frequency was set to 60–70 rpm. Participants were strongly encouraged to produce maximal effort. Respiratory gas exchange variables were measured throughout the test using breath-by-breath mode with data being recorded in 10 s intervals. Subjects breathed through a facemask. Oxygen consumption, carbon dioxide output and minute ventilation were continuously measured using a portable open-air spirometry system (MetaMax I, Cortex, Leipzig, Germany). The analyzer was calibrated with gases of known concentration before the test according to the manufacturer’s guidelines. All data were calculated by means of computer analysis using standard software (MetaMax-Analysis 3.21, Cortex, Leipzig, Germany). Peak oxygen consumption (VO_2_peak; L/min) was measured and aerobic performance was defined as VO_2_peak per kilogram of LBM (mL/min/kg LBM) [27]. 

#### 2.2.3. Blood Analysis

Venous blood samples were drawn between 8:00 and 9:00 a.m. after an overnight fast from an antecubital vein with the participants sitting in an upright position [19]. Blood serum was separated and frozen at −80 °C for further analyses. Estradiol and insulin-like growth factor-1 (IGF-1) were analyzed using Immulite 2000 (DPC, Los Angeles, CA, USA). The intra- and inter-assay CVs for estradiol were less than 7%. The intra- and inter-assay CVs for IGF-1 were lower than 6%. Leptin was determined by Evidence^®^ Biochip Technology (Randox Laboratories Ltd., Crumlin, UK) with intra- and inter-assay CVs of 4.6% and 6.0% [19]. 

### 2.3. Statistical Analysis

Data analysis was performed using the SPSS software version 21.0 package for Windows (Chicago, IL, USA). Standard statistical methods were used to calculate means and standard deviations (±SD). For sample size calculation, the SD for bone mineral values based on previously published data [7,14] was used. Accordingly, with a 0.80 chance (80% power) to detect a difference at 0.05, we needed at least 19 participants in each group [29]. Evaluation of data normality was performed with the Kolmogorov–Smirnov method. Data that were not normally distributed were logarithmically transformed prior to analyses to approximate a normal distribution. Statistical comparisons between the groups were made using one-way analysis of variance (ANOVA) and a Bonferroni post hoc test. Effect size (ES, eta squared) thresholds of 0.01, 0.06 and 0.14 were used to identify a small, moderate and large effect from the ANOVA analyses to define the magnitude of the effect [30]. Pearson correlation coefficients were calculated to assess linear relationships. Multiple regression analysis was performed to identify the effects of IGF-1, estradiol, and leptin together with body fat%, appendicular muscle mass, age, age at menarche and training volume on measured aBMD and BMC values [10]. Stepwise and R^2^ methods were used for model selection [28]. Model R^2^ and partial R^2^ of the individual model parameters are reported, and R^2^ is the coefficient of determination and represents the portion of total variation attributable to the variables in the model [31]. The level of significance was set at *p* < 0.05.

## 3. Results

Mean age, body height, body mass and BMI were not different among RG, SW and UC groups (Table 1). Age at menarche was higher, and body fat% and FM were lower in RG athletes in comparison with SW and UC groups (all *p* < 0.0001). Body lean mass and appendicular muscle mass were not different (*p* > 0.05) between different athletic activity groups but were higher compared with the UC group. In addition, VO_2_peak/LBM in RG athletes was lower and higher in comparison with SW and UC groups, respectively (all *p* < 0.0001). No differences (*p* > 0.05) in weekly training volume were observed between RG (17.6 ± 5.3 h/week) and SW (16.1 ± 6.9 h/week) athletes (Table 1). Circulating IGF-1 concentration was significantly higher in SW athletes in comparison with RG athletes and the UC group, while leptin levels were higher (*p* < 0.0001) in both athletic groups in comparison with the UC group. No differences in estradiol values were observed between the studied groups (Table 1).

**Figure 1:**
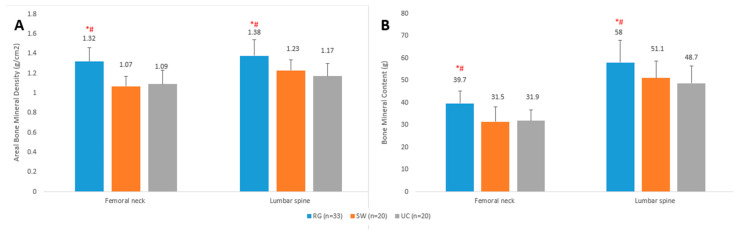
Mean (±SD) areal bone mineral density (**A**) and bone mineral content (**B**) values of rhythmic gymnasts (RG), swimmers (SW) and untrained controls (UC). * Significantly different from untrained controls; *p* < 0.05. ^#^ Significantly different from swimmers; *p* < 0.05.

Measured aBMD and BMC values were higher in RG athletes compared with SW and UC groups (*p* < 0.05), while no differences (*p* > 0.05) in bone mineral values were seen between SW and UC groups (Figure 1).

Table 2 presents correlation coefficients of bone mineral values with blood biochemical, training and body composition variables in RG, SW and UC groups. In RG athletes, significant correlations of estradiol, body fat% and appendicular muscle mass with FN aBMD were observed. In addition, muscle mass was related to FN BMC, body fat% to LS aBMD and appendicular muscle mass together with body fat% were related to LS BMC values in RG athletes. In the SW group, estradiol, age at menarche and muscle mass were correlated with LS BMC values. In the UC group, all measured aBMD and BMC values were only correlated with appendicular muscle mass (Table 2).

Multiple regression models revealed that IGF-1 was the predictor of bone mineral values only in the RG group and explained 14% of the variance (R^2^ × 100) in LS aBMD (*p* = 0.031) (Table 3), while the strongest predictor for FN BMC was appendicular muscle mass (by 28%; *p* = 0.006) followed by training volume (by 9%; *p* = 0.039) in the RG group. In the SW group, the strongest predictor for LS BMC was age at menarche (by 27%; *p* = 0.001), followed by estradiol (by 21%; *p* = 0.001) and appendicular muscle mass (by 20%; *p* = 0.004). In the UC group, appendicular muscle mass was the only predictor for FN aBMD (by 20%; *p* = 0.029), FN BMC (by 53%; *p* < 0.0001), LS aBMD (19%; *p* = 0.031) and LS BMC (by 25%; *p* = 0.014) (Table 3).

## 4. Discussion

The present investigation was undertaken to examine the effect of heavy athletic activity on bone mineral accrual in well-trained adolescent female athletes with different mechanical loading patterns. Another aim was to study the possible associations of bone mineral values with body composition, blood hormone and training load variables in the studied groups. The main finding of the present study was that all measured bone mineral values were significantly higher in RG athletes compared with SW and UC groups, which did not differ in measured aBMD and BMC variables. These results demonstrate that heavy gymnastics activity in adolescent female RG athletes positively influenced bone mineral accrual despite lowered body FM values, while heavy swimming activity in adolescent SW athletes was neutral for bone mineral accrual in adolescent SW athletes. It appeared that weekly training volume together with appendicular muscle mass was a significant predictor of FN BMC values in RG athletes, while age at menarche and estradiol concentrations together with appendicular muscle mass were important predictors of LS BMC values in SW athletes. Appendicular muscle mass alone was the only predictor of all measured bone mineral values in the UC group. These results suggest that bone mineral accumulation is affected by specific athletic activity in the measured cortical bone of the skeleton in athletes undertaking weight-bearing activities, while hormone values influence trabecular bone of the skeleton in athletes undertaking non-weight-bearing activities, and muscle mass alone influenced all measured bone mineral values in the UC group.

One of the main findings of the present study was that heavy participation in weight-bearing athletic activity with a mean weekly training volume of 17.6 ± 5.3 h seems to be able to affect bone mass accumulation directly through specific mechanical impacts of gymnastics training and/or indirectly through appendicular muscle mass in a relatively homogeneous group of adolescent RG athletes. Higher aBMD and BMC values in RG athletes were observed in measured both cortical and trabecular sites of the skeleton in comparison with other groups. These results confirm previous investigations with heterogeneous groups of RG athletes who presented rather wide age ranges from 10 to 18 years [7,32], but also provide additional information, since well-trained adolescent RG athletes with a narrow age range have not been extensively studied. Specifically, Munoz et al. [33] found that well-trained adolescent RG athletes had higher cortical aBMD at the FN site of the skeleton. It has been suggested that there might not be enough mechanical loading to produce additional bone mineralization in other skeletal sites than FN of the skeleton the specific gymnastics training of RG athletes [7], while the results of our study demonstrated that that of the LS site of the skeleton was also higher as a result of heavy athletic activity in adolescent RG athletes. In accordance with our results, it has also been suggested that bone mass acquisition may increase in RG athletes after 14 years and the difference between RG and UC groups may be evident only after 16 years of age [7]. Interestingly, although the SW group trained more than 16 h per week, they presented a profile of bone mineral acquisition similar to that of the UC group, demonstrating that heavy athletic swimming activity has a neutral effect on areal bone mineral values in female adolescent SW athletes, despite the large amount of muscle contractions during swimming training. However, it has also been argued that training load may even negatively affect bone mineral accrual in SW athletes, independently of the positive effects of muscle mass on bone mineral density in adolescent swimmers [34]. It has further been suggested that when SW athletes reach adulthood, they may present lower bone mineral values in comparison with weight-bearing athletes, including RG athletes [13]. The non-osteogenic effect of athletic swimming activity observed in our study is in line with previous studies in pubertal [4] and adult [14] female swimmers. Accordingly, swimming may not be one of the athletic activities to practice in order to increase the development of bone mineral values due to the hydrostatic forces against gravity and lack of impact characteristics of this specific sport event [14].

It has been suggested that training volume is associated with direct and indirect pathways to bone mineral adaptations in adolescent athletes [8,16,35]. Accordingly, prescribing athletic activity models based on training volume could be a valuable strategy to improve bone health values in adolescents [8]. In our study, weekly training volume was a significant predictor of areal bone mineral values in RG but not in SW athletes. These results demonstrate that weight-bearing mechanical activity of athletic gymnastics activity directly influences cortical bone at the FN site of the skeleton in well-trained adolescent RG athletes. In accordance, a dose–response relationship between gymnastics exposure (i.e., hours and years of training) and bone mineral accrual has also been suggested in other studies [15,36], and the positive effect of the mechanical activity of gymnastics training on the skeleton is more pronounced in cortical bone compared with trabecular bone, which is more prone to changes in specific hormone concentrations [6]. In contrast, prolonged practice of athletic swimming activity may even be negatively related to bone mineral accrual in adolescents [13,37]. Accordingly, it is also important to know which specific mechanical activity has beneficial effects on bone mineral accrual in female athletes during adolescence. These results further demonstrate that regular participation in weight-bearing athletic activities during adolescence is beneficial for bone mineral accrual. In addition, an indirect pathway for bone mineral adaptations in adolescent RG athletes includes whole-body and regional lean soft tissue mass [8,9], which has been reported to be associated with training volume in adolescent athletes [8,12,28,38]. In accordance, appendicular muscle mass was associated with areal bone mineral values in all studied groups in the present study. Muscle mass has been suggested to be the main pathway through which any physical exercise including athletic activity can improve part of the osteogenic process in adolescents during maturation [34,39,40]. However, although appendicular muscle mass was related to bone mineral values in SW athletes and they had the highest values of aerobic performance, their bone mineral values were lower than in the RG group. These results would suggest that one of the most important pathway to increase bone mineral adaptations during adolescence is a specific weight-bearing mechanical activity of athletic gymnastics activity, as also demonstrated by the significant association between weekly training volume and bone health in adolescent RG athletes.

It has been reported that while cortical bone, such as the FN site of the skeleton, is more influenced by specific mechanical patterns of the athletic activity, trabecular bone, such as the LS site of the skeleton, is more influenced by increased energy expenditure together with hormonal regulation caused by the heavy athletic activity [6,18]. In our study, LS aBMD was influenced by circulating IGF-1 levels in adolescent RG athletes. In accordance, IGF-1 as a myokine mediates muscle mass and bone mineral metabolism [41], not only during growth and maturation but also through the entire life span [42]. In addition, estradiol has been reported to correlate with IGF-1 in adolescent RG athletes [4], which positively affect bone turnover by stimulating osteoblast proliferation and differentiation [43]. Furthermore, it has been suggested that the indices of the IGF-1 axis may serve as surrogate markers of bone mineral gain in RG athletes during growth and maturation [44]. In contrast, leptin as an adipokine did not influence bone mineral accrual in any group of the studied adolescent females, although leptin and FM values were lower in both athletic groups. Previously, it has been reported that leptin is positively correlated with FM and aBMD values in lean UC girls with different maturation levels, while the impact of lowered leptin on bone mineral accrual in the presence of elevated energy expenditure and reduced FM remains unclear in maturing female athletes [6]. Accordingly, further studies are needed to assess specific myokine and adipokine values in order to better understand their possible role in bone mineral accrual in heavily exercising adolescent female athletes with different loading patterns.

There are some limitations in our investigation that should be acknowledged. Firstly, the relatively small sample size, determined by the small number of well-trained athletes, which also represent a very specific group of adolescent females (Caucasian, narrow age range, similar body composition). However, the number of participants was comparable to previous similar studies in this area [7,12,14,32]. Secondly, the cross-sectional design of the investigation, which cannot determine the cause-effect relationships. On the other hand, the present study has also several strengths. We present data for the healthy population with well-defined body composition and specific mechanical loading patterns on bone in a specific pediatric population. In addition, appendicular muscle mass is included in this study as it has a positive effect on bone, while few studies have reported lean soft tissue values before. The results of our study also suggest that different physiological systems integrate their dynamics to increase bone mineral accrual and maintain health in adolescent female athletes [45]. Finally, the study participants had years of practice with long weekly hours of training, which has not been taken into account in many studies, when investigating bone health in adolescent female athletes.

## 5. Conclusions

Adolescent RG athletes undertaking specific weight-bearing athletic activity present higher areal bone mineral values in comparison with SW and UC groups, while non-weight-bearing activity has a neutral effect on bone mineral accrual in adolescent SW athletes. Specific training volume together with appendicular muscle mass influenced cortical bone development at the FN site of the skeleton in RG athletes, while hormonal values influenced trabecular bone development at the LS site of the skeleton in both athletic groups with different loading patterns. It could be suggested that swimming training in adolescent female SW athletes should be combined with specific weight-bearing, impact or strength activities to further influence bone mineral accrual, as swimming does not seem to be an osteogenic sport. Finally, appendicular muscle mass was the only predictor of all measured bone mineral values in the UC group.

## Figures and Tables

**Table 1:** Mean (±SD) characteristics of rhythmic gymnasts (RG), swimmers (SW) and untrained controls (UC).

Variable	RG (*n* = 33)	SW (*n* = 20)	UC (*n* = 20)	*p* Value	ES
Age (yrs)	16.0 ± 1.2	15.7 ± 0.9	16.5 ± 1.6	0.134	0.06
Age at menarche (yrs)	13.6 ± 1.2 *^#^	12.7 ± 1.1	12.5 ± 0.7	<0.0001	0.22
Body height (cm)	166.8 ± 5.3	169.8 ± 4.6	166.8 ± 5.0	0.081	0.07
Body mass (kg)	55.7 ± 7.0	59.7 ± 3.6	58.4 ± 7.4	0.071	0.07
BMI (kg/m^2^)	20.0 ± 2.0	20.7 ± 1.0	21.0 ± 2.2	0.137	0.06
Body fat %	19.5 ± 5.7 *^#^	24.2 ± 3.8 *	30.4 ± 6.2	<0.0001	0.42
Body fat mass (kg)	11.2 ± 4.3 *^#^	14.5 ± 2.5 *	17.8 ± 4.8	<0.0001	0.32
Body lean mass (kg)	42.2 ± 4.1 *	42.8 ± 3.1 *	37.7 ± 3.7	<0.0001	0.25
Appendicular muscle mass (kg)	19.4 ± 2.1 *	19.4 ± 2.0 *	16.8 ± 1.6	<0.0001	0.27
VO_2_peak/LBM (mL/min/kg)	53.6 ± 7.7 *^#^	64.9 ± 5.8 *	48.4 ± 5.6	<0.0001	0.48
Training volume (h/week)	17.6 ± 5.3 *	16.1 ± 6.9 *	2.1 ± 1.3	<0.0001	0.64
IGF-1 (ng/mL)	283.3 ± 55.6	305.6 ± 44.6 *	250.7 ± 68.7	0.012	0.12
Estradiol (pmol/L)	187.3 ± 56.8	206.5 ± 53.6	171.3 ± 69.3	0.183	0.05
Leptin (ng/mL)	1.2 ± 0.6 *	1.6 ± 0.9 *	3.7 ± 2.6	<0.0001	0.33

Note: ES, effect size (eta squared); VO_2_peak/LBM, peak oxygen consumption per kg lean body mass; IGF-1, insulin-like growth factor-1. * Significantly different from untrained controls, *p* < 0.05; ^#^ significantly different from swimmers, *p* < 0.05.

**Table 2:** Pearson correlation coefficients of bone mineral values with blood biochemical, training and body compositional variables.

Variable	FN aBMD (g/cm^2^)			FN BMC (g)			LS aBMD (g/cm^2^)			LS BMC (g)		
	RG	SW	UC	RG	SW	UC	RG	SW	UC	RG	SW	UC
IGF-1 (ng/mL)	0.17	0.22	0.32	0.09	−0.08	0.18	0.24	−0.22	0.13	0.11	−0.04	0.16
Estradiol (pmol/L)	0.35 *	−0.25	0.19	0.10	−0.27	−0.01	0.16	−0.13	−0.10	0.30	−0.47 *	−0.02
Leptin (ng/mL)	0.20	−0.07	0.29	0.18	0.14	−0.25	0.21	0.23	0.32	0.23	0.01	−0.32
Age at menarche (yrs)	−0.26	−0.15	0.19	−0.24	0.05	0.24	−0.03	−0.23	0.28	−0.22	−0.56 **	−0.20
VO_2_peak/LBM (mL/min/kg)	−0.05	0.35	0.06	0.04	−0.29	0.06	0.06	0.30	0.35	0.02	0.02	0.29
Training volume (h/week)	−0.14	0.04	0.39	0.18	−0.27	0.11	−0.15	0.11	0.18	−0.22	−0.22	0.09
Body fat%	0.38 *	0.03	−0.35	0.29	0.14	−0.17	0.48 *	0.23	−0.01	0.53 **	0.10	−0.07
Appendicular muscle mass (kg)	0.40 *	0.36	0.49 *	0.59 **	0.10	0.75 **	0.30	0.22	0.48 *	0.41 *	0.46 *	0.54 *

Note: * *p* < 0.05; ** *p* < 0.01.

**Table 3:** Multiple regression models.

Variables ^a^	B Coefficient ± SE	Partial *R*^2^	*p* Value	Model Summary
Rhythmic gymnasts				
FN aBMD	-	-	-	-
FN BMC				*R*^2^ = 0.37; *p* = 0.001
Appendicular muscle mass	1.172 ± 0.389	0.28	0.006	
Training volume	0.345 ± 0.159	0.09	0.039	
LS aBMD				*R*^2^ = 0.14; *p* = 0.034
IGF-1	0.001 ± 0.001	0.14	0.031	
LS BMC	-	-	-	-
Swimmers				
FN aBMD	-	-	-	-
FN BMC	-	-	-	-
LS aBMD	-	-	-	-
LS BMC				*R*^2^ = 0.68; *p* < 0.0001
Age at menarche	−3.660 ± 0.932	0.27	0.001	
Estradiol	−0.074 ± 0.019	0.21	0.001	
Appendicular muscle mass	1.711 ± 0.503	0.20	0.004	
Untrained Controls				
FN aBMD				*R*^2^ = 0.20; *p* = 0.029
Appendicular muscle mass	0.042 ± 0.018	0.20	0.029	
FN BMC				*R*^2^ = 0.53; *p* < 0.0001
Appendicular muscle mass	2.284 ± 0.484	0.53	<0.0001	
LS aBMD				*R*^2^ = 0.19; *p* = 0.031
Appendicular muscle mass	0.038 ± 0.016	0.19	0.031	
LS BMC				*R*^2^ = 0.25; *p* = 0.014
Appendicular muscle mass	2.602 ± 0.961	0.25	0.014	

Note: ^a^ Variables tested in the model: IGF-1, estradiol, leptin, body fat%, appendicular muscle mass, age, age at menarche and training volume.

## Data Availability

The data presented in this study are available on a request from the corresponding author for researchers who meet the criteria for access to confidential data.
